# The Human Brain Representation of Odor Identification in Amnestic Mild Cognitive Impairment and Alzheimer's Dementia of Mild Degree

**DOI:** 10.3389/fneur.2020.607566

**Published:** 2021-01-13

**Authors:** Grete Kjelvik, Hallvard R. Evensmoen, Thomas Hummel, Knut Engedal, Geir Selbæk, Ingvild Saltvedt, Asta K. Håberg

**Affiliations:** ^1^Norwegian National Advisory Unit on Ageing and Health (Ageing and Health), Tønsberg, Norway; ^2^Department of Radiology and Nuclear Medicine, St. Olavs Hospital (Norwegian National Advisory Unit for Functional Magnetic Resonance Imaging), University Hospital of Trondheim, Trondheim, Norway; ^3^Department of Neuromedicine and Movement Science, Norwegian University of Science and Technology, Trondheim, Norway; ^4^Smell and Taste Clinic, Department of Otorhinolaryngology, Technische Universität Dresden, Dresden, Germany; ^5^Institute of Clinical Medicine, University of Oslo, Oslo, Norway; ^6^Department of Geriatric Medicine, Oslo University Hospital, Oslo, Norway; ^7^Department of Geriatrics, Clinic of Medicine, St. Olavs Hospital, University Hospital of Trondheim, Trondheim, Norway

**Keywords:** smell, olfaction, neurodegenaration, central nervous system (CNS), cognition

## Abstract

**Background:** Odor identification (OI) ability is a suggested early biomarker of Alzheimer's disease. In this study, we investigated brain activity within the brain's olfactory network associated with OI in patients with amnestic mild cognitive impairment (aMCI) and mild Alzheimer's dementia (mAD) to uncover the neuronal basis of this impairment.

**Materials and Methods:** Patients with aMCI (*n* = 11) or mAD (*n* = 6) and 28 healthy older adults underwent OI functional MRI (fMRI) at 3T, OI, odor discrimination, and cognitive tests and apolipoprotein-e4 (APOE4) genotyping. Eleven patients had cerebrospinal fluid (CSF) analyzed. Those with aMCI were followed for 2 years to examine conversion to dementia.

**Results:** The aMCI/mAD group performed significantly worse on all OI tests and the odor discrimination test compared to controls. The aMCI/mAD group had reduced activation in the right anterior piriform cortex compared to the controls during OI fMRI [Gaussian random field (GRF) corrected cluster threshold, *p* < 0.05]. This group difference remained after correcting for age, sex education, and brain parenchymal fraction. This difference in piriform activity was driven primarily by differences in odor discrimination ability and to a lesser extent by OI ability. There was no group by odor discrimination/identification score interaction on brain activity. Across both groups, only odor discrimination score was significantly associated with brain activity located to the right piriform cortex. Brain activity during OI was not associated with Mini Mental Status Examination scores. At the group level, the aMCI/mAD group activated only the anterior insula, while the control group had significant activation within all regions of the olfactory network during OI fMRI. There was no association between brain activity during OI fMRI and total beta-amyloid levels in the CSF in the aMCI/mAD group.

**Conclusion:** The OI impairment in aMCI/mAD patients is associated with significantly reduced activity in the piriform cortex compared to controls. Activation of downstream regions within the olfactory network is also significantly affected in the aMCI/mAD group, except the anterior insula, which is impinged late in the course of Alzheimer's disease. OI tests thus reflect Alzheimer's disease pathology in olfactory brain structures.

## Introduction

Odor identification (OI) is considered an early biomarker of Alzheimer's disease (1). Patients with amnestic mild cognitive impartment (aMCI) who are at risk of developing AD and patients with Alzheimer's dementia (AD) have a specific impairment in OI but also display reduced odor detection and odor discrimination abilities (2–6). As healthy older adults also experience a decline in odor detection and discrimination, but not a similar reduction in OI, OI is considered to separate people with aMCI or AD from older people with intact cognition (5, 7). Importantly, OI ability is shown to predict both a later diagnosis of MCI in healthy older adults and conversion from MCI to dementia (8, 9). Hence, OI testing can be used as an inexpensive and non-invasive supplement in the clinical evaluation of suspected AD (1, 10). OI's utility as a clinical tool and biomarker of dementia risk depends on a better understanding of the neuronal correlates underlying OI impairment in the early symptomatic phase of Alzheimer's disease.

In the brain, olfactory stimuli are processed in the olfactory network (ON), which includes the primary (piriform cortex, entorhinal cortex, amygdala) and secondary (hippocampus, thalamus, orbitofrontal cortex, and insula) olfactory regions (11, 12). These regions are affected in a sequential manner by tau and beta-amyloid pathologies during the course of Alzheimer's disease. In the early symptomatic period of Alzheimer's disease when aMCI presents, tau pathology is found in the entorhinal and piriform cortices, the amygdala, and to a limited degree in the hippocampus, while β-amyloid is present in the orbitofrontal cortex. As the disease develops and dementia is diagnosed, tau pathology has spread to the anterior insula while β-amyloid plaques can be detected in the amygdala and allocortical structures (e.g., entorhinal and piriform cortex, hippocampus) (13–17).

Previous functional MRI (fMRI) studies of olfaction in patients with MCI and AD have focused on aspects of odor perception. They report greatly reduced whole-brain activity to smelling (18, 19) as well as fewer activated voxels or lower fMRI signal in regions of interest in the piriform cortex/primary olfactory cortex and/or hippocampus during smelling, applying uncorrected statistical approaches (19, 20). Moreover, impaired cross-adaptation (19) and habituation (21, 22) of the fMRI signal have been demonstrated in regions of interest in the piriform cortex using uncorrected statistics in patients with AD and MCI. Taken together, the knowledge is sparse with regard to the neural substrates of OI impairment in patients with aMCI and dementia due to Alzheimer's disease (AD).

We investigated OI using fMRI at 3T in patients with aMCI and Alzheimer's dementia of mild degree (mAD) compared to healthy older adults. To this end, we used an OI paradigm that provides robust activation within all regions of the ON (23). We hypothesized reduced OI fMRI activity in patients with aMCI/mAD in the ON regions affected early in the course of Alzheimer's disease, i.e., the entorhinal and piriform cortices, and amygdala, while the insula would be less affected due to the later occurrence of Alzheimer's disease pathology in this region.

## Materials and Methods

### Participants

Nineteen patients (between 65 and 81 years) from the Memory Clinic, Geriatric Department, St. Olav's Hospital, Trondheim, Norway, agreed to participate and were MRI compatible. Inclusion criteria were a diagnosis of aMCI probably caused by Alzheimer's disease or mAD according to a comprehensive clinical assessment at the time of inclusion, age >55 years, and MRI compatibility. Patients were examined and diagnosed by an experienced geriatrician according to the research criteria of the International Classification of Diseases (ICD-10) and the National Institute of Neurological and Communicative Disorders and Stroke and the Alzheimer's Disease and Related Disorders Association (NINCDS-ADRDA) (18, 24). Patients fulfilling the accepted US National Institute on Aging–Alzheimer's Association (NIA-AA) diagnostic criteria for aMCI were also included (25, 26). The examination encompassed a diagnostic workup with medical history obtained from both patient and their caregivers and clinical examination including neurological examination, cardiovascular status, and cerebral MRI. Cognitive function was assessed with the Mini Mental Status Examination (MMSE), Trail Making Tests A and B (TMT-A and TMT-B, respectively), and the Ten-Word Test (TWT) from the Consortium to Establish a Registry for Alzheimer's disease (CERAD) (27). Handedness was determined with the Edinburgh Handedness Inventory (28). Ethylenediaminetetraacetic acid (EDTA) blood samples were obtained to determine apolipoprotein-e4 (APOE4) allele status. In 11 patients, the cerebrospinal fluid (CSF) biomarker β-amyloid was analyzed.

Two patients were excluded; one because of an olfactory meningioma uncovered in this study, and the second patient developed late-onset bipolar depression, which was considered the cause of the initial aMCI diagnosis. Thus, 17 patients, 11 (four men and seven women) with aMCI and six (four men and two women) with mAD, were included. At 6 months' follow-up, 13 of the aMCI patients had progressed to dementia due to AD, while four were still diagnosed as aMCI. After 2 years, two of those with aMCI had converted to dementia, while two remained aMCI. These two participants with aMCI after 2 years of follow-up had typical symptoms of Alzheimer's disease. All included aMCI/mAD patients were right-handed as determined with the Edinburgh Handedness Inventory, with a mean score of 93.0 ± 9.7.

A control group of 35 healthy adults was recruited from senior citizen centers, advertisements, and personal networks. MRI compatibility was an inclusion criterion. In total, 28 controls (14 men and 14 women) between 55 and 81 years were included. The participants in the control group performed the same cognitive test battery (MMSE, TMT-A, TMT-B, and TWT) plus the Edinburgh Handedness Inventory, and blood was obtained for APOE4 allele testing. Seven controls were excluded due to technical problems during MRI scanning. Of the control participants included, 93% were right-handed and 7% were left-handed with a mean Edinburgh Handedness Inventory score of 82.4 ± 29.

The sample size for this fMRI study was determined based on analyses of pilot data and data from previous fMRI studies we have conducted. In addition, we performed an *a priori* power analysis using G^*^Power (https://www.psychologie.hhu.de/arbeitsgruppen/allgemeine-psychologie-und-arbeitspsychologie/gpower.html). With an assumed medium effect size of 0.55, 80% power, and alpha = 0.05, the sample size of *n* = 21 per group was estimated.

At the time of the MRI, all participants completed a self-evaluation form of previous and present smoking habits. Anterior rhinoscopy was performed in all participants, and they were systematically checked for a history of olfactory, nasal, and/or respiratory problems (trauma, septum deviation, nasal/sinus surgery, hypertrophic rhinitis, drug-induced rhinitis, cold, upper respiratory tract infection, acute or chronic sinusitis, nasal tumors, Sjøgren's syndrome, or nasal polyposis). Five participants had seasonal pollen/grass allergy, but none had active allergy when the experiment was conducted.

The study was approved by the Regional Committee of Medical Research Ethics (REC-mid Norway) and the Norwegian data inspectorate. All participants had the capacity to consent to participation, and they gave written informed consent after the procedure had been carefully explained and after they had the opportunity to ask questions about the research.

### MRI

MRI examinations were performed on one Siemens Trio 3T system (Siemens, Erlangen, Germany) equipped with a 12-channel head coil. Foam pads were used to minimize head motion. The scan protocol included a high-resolution T1-weighted three-dimensional (3D) MPRAGE sequence (196 slices; TE 30 ms; TR 2,300 ms; isotropic voxels of 1 mm^3^), followed by a T2-weighted image series of the sinuses and nasal cavity (40 slices; TE 77 ms; TR 4,290 ms; slice thickness 2 mm). The latter sequence was used to exclude individuals with pathology in the nasal cavity, sinuses, and/or olfactory bulbs and tract. If the participant did not have a structural pathology, the nasal mask (Respironics, ScanMed AS, Norway) was put on before fMRI. The subject was repositioned in the scanner, and a new scout image for positioning of the fMRI scans was obtained.

Two OI fMRI runs were performed using a T2^*^-weighted blood oxygen level-dependent (BOLD) sensitive, single echo-planar imaging (EPI) pulse sequence [47 slices; TE 30 ms; TR 2,600 ms; field of view (FoV) 230 mm, giving a resolution of 3 × 3 × 3 mm; acquisition matrix 80 × 80]. Each fMRI run consisted of 265 volumes plus three dummy scans for magnetization stabilization, giving a total acquisition time of 12 min for each run. The slices were angled as perpendicular to the long axis of the hippocampus as possible and the slice package anterior border was the frontal pole to optimize imaging of the ON, which is prone to susceptibility artifacts (29).

### The Olfactometer

**Figure 1:**
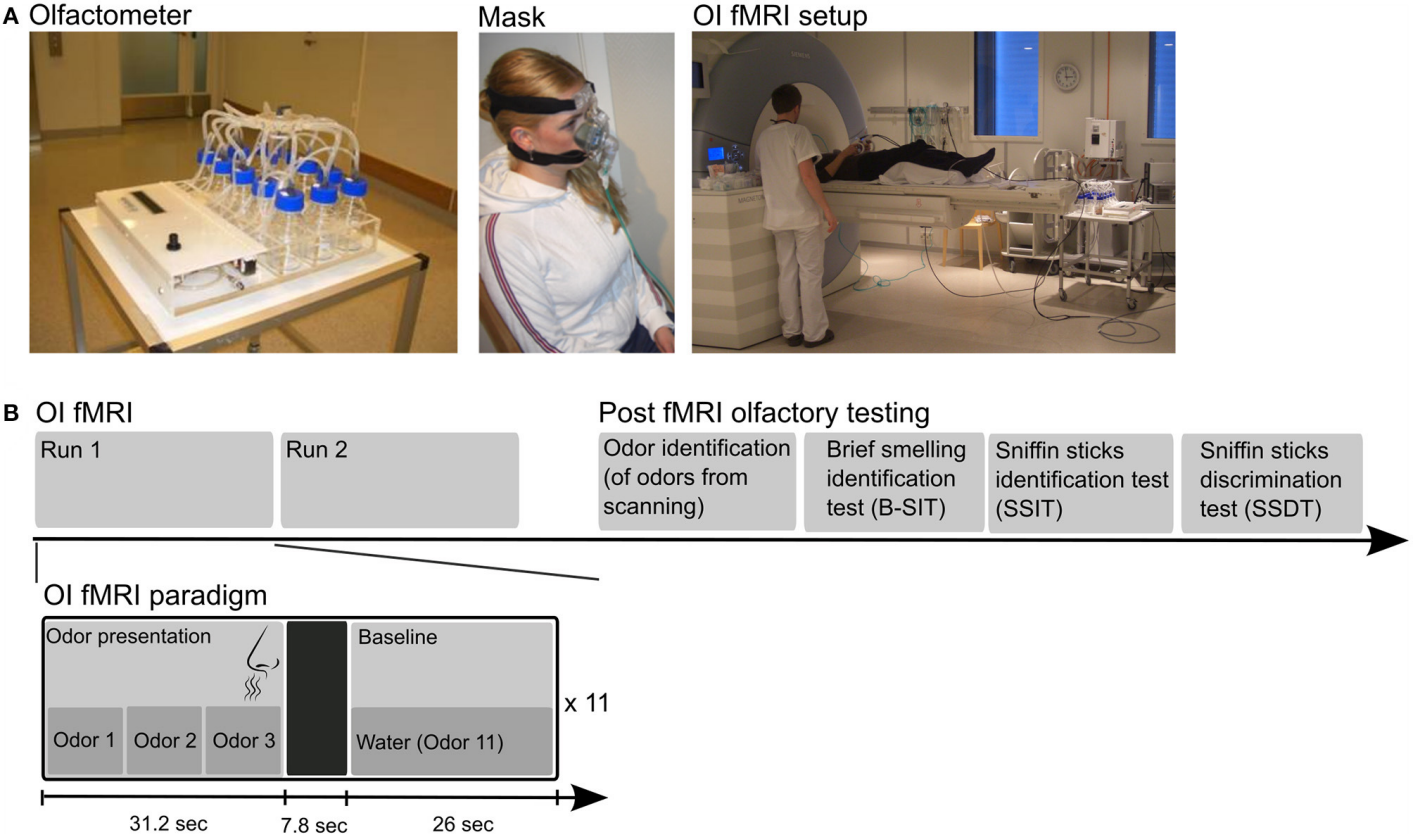
The top row **(A)** displays photos of the automated olfactometer to the left, followed by a person wearing the olfactory mask in the middle, and the setup in the scanner room on the right. **(B)** The lower row shows the design of the fMRI experiment. The experiment consisted of two fMRI runs with odor identification (OI) followed by psychophysical olfactory tests completed after MRI scanning. The fMRI paradigm was a mixed block (OI state)–event (successful OI) design (bottom row). Each run consisted of 11 olfactory blocks, and each olfactory block consisted of three odors in a random order, followed by a 7.8-s null event period (to ensure removal of scented air) and a non-odor baseline condition (water). The persons in the photos have provided consent for publication.

Odor stimuli were presented with a custom-built MRI-compatible, automated olfactometer built by an engineer at the Norwegian University of Science and Technology (NTNU) based on modifications of earlier MRI-compatible olfactometers (30–32). The olfactometer has 14 glass chambers for deposition of liquid odors and allows the odor stimuli in the chambers to be delivered into the nasal mask in a preprogrammed and timed order, i.e., each odor is presented at a certain time for a certain duration (Figure 1). The olfactometer was positioned 2.25 m from the magnet's isocenter during fMRI. Medical air flowing at a rate of 12 L/min went into the odor-filled chambers, allowing the odors to be released. From each chamber, the odor was conveyed *via* a separate tubing into the main Teflon tubing entering the nasal mask (Figure 1). Since odors were released in the air into the mask and not delivered directly into the nostrils, body temperature heated the incoming scented air (33). An additional hole at the superior end of the mask was connected to the hospital's gas evacuation system *via* tubings to ensure continuous airflow and removal of scented air. All tubings were made of very low adsorbent material (Teflon fluorinated ethylene propylene) to minimize absorption of odor molecules into the tubes (33). The olfactometer was started by the experimenter using a remote control from the scanner operating room exactly at the time of initiation of fMRI scanning.

### Olfactory fMRI Paradigm

The participants performed an OI task during fMRI based on Kjelvik et al. (23). They were told not to sniff, just breath regularly throughout the experiment, and let the air with the odor pass over and into the nose. The participants were asked to identify the odors, and in case they were confident of correct OI, to press a response button (NordicNeuroLab AS, Bergen, Norway). This design was deemed feasible for older adults based on OI fMRI pilot studies that showed that collecting responses using, for instance, forced choice between odor names presented on a screen during fMRI was too complicated for this group.

The participants were familiarized with the odor task, breathing, the mask, and the response button before scanning. Scanning started when participants performed the task correctly. The participants were informed that they would be asked to identify the same odors after scanning.

The OI fMRI paradigm was a mixed block (OI state)–event (self-reported successful OI) design. Ten odor chambers were filled with liquid odorants, and one chamber was filled with water. Two milliliters of odor-liquids were used for each of the 10 odors; lemon, chocolate toffee, musk, anise, banana, and rose (Stockholm's Essence Fabric, Wallinggatan 14, 111 24 Stockholm, Sweden; www.essencefabriken.se), vanillin and apple (Sigma Aldrich, Germany), and fresh coffee and cinnamon from local suppliers were in water solutions. Each odorant was presented for 10.4 s to ensure that at least one breathing cycle was completed within the stimulus' duration (Figure 1) (23). Each olfactory block consisted of three odors in a random order; e.g., lemon–coffee–cinnamon. The total duration of an odor block was 31.2 s. A total of 22 olfactory blocks (i.e., 66 odor exposures) were presented to each participant across two runs. Each of the 10 odors was presented between six and 10 times in a pseudo-random manner. Water was used as the baseline non-odor condition and presented in blocks of 26 s between the olfactory blocks. A 7.8-s period following each odor block was used as a null event to ensure removal of scented air (Figure 1). The participants were asked to identify the odors during OI fMRI, and in case they were confident they were correct, to press the response button.

Between participants, the olfactometer was carefully cleaned, and the nasal mask was disinfected with PeraSafe (Puls AS, Oslo, Norway). The short tubings from each chamber were removed, and new tubings were added after each day of scanning, and new odor-liquids were used at the day of scanning. After each experiment, medical air from the hospital's gas provision system was used for 10 min to clean the long tubings and the olfactometer.

### Post-scan Assessment

After fMRI acquisition, the participants were presented with an OI task with the same 10 odors as during fMRI. The odors were presented in a random order, each in 1-ml liquid solutions in glass bottles. Participants were asked to identify the odors first spontaneously, and then with forced multiple choice with four alternatives. Subsequently, two standard clinical OI tests were used to evaluate the participants' OI abilities: the Brief Smell Identification Test (B-SIT; Sensonics Inc., Haddon Heights, NJ, USA) and the Sniffin' Sticks Identification Test (SSIT, Burghart Messtechnik, Wedel, Germany). B-SIT is a scratch and sniff test with 12 microcapsulated odorants and a forced multiple choice between four items per odorant. The SSIT consists of 16 penlike odor-dispensing devices, with common odors, and uses forced multiple choice between four items per test odorant. The participants were allowed to sniff at the Sniffin' Sticks pens once or twice for 3–4 s. In both tests, the alternatives were given orally twice from the experimenter; in addition, the participants read the alternatives themselves on a card presented with the odor. The participants were told to give an answer for all odors, even if they did not smell anything, to make the tests valid. No feedback was given during the administration of the tests. The Sniffin' Sticks Discrimination Test (SSDT, Burghart Messtechnik, Wedel, Germany) was performed to evaluate odor discrimination abilities. The SSDT was completed in 13 of the patients (nine aMCI and four mAD) due to fatigue in the others. The SSDT consists of 16 triplets, where two pens have the same smell, while one of the three pens contains a different odor. Participants were asked to identify the pen that had the different odor and were blindfolded because the pens were color-coded. Participants were asked to choose one of the three pens of the triplet even if they did not perceive or recognize a difference between the odors. In each olfactory test, correctly identified odors received one point, giving a possible score range of 0–12 points for B-SIT, 0–16 for SSIT, and 0–16 for SSDT.

### Statistical Analysis of Demographic and Behavioral Data

Statistics were performed using SPSS version 26 (SPSS, IBM). Characteristics of the control group and the aMCI/mAD group, as well as performance on cognitive tests, were compared using Student's *t*-tests. Differences with regard to cognitive test scores between the aMCI and mAD groups were also assessed with Student's *t*-tests. Group differences on psychophysical tests (free recall, multiple choice test, B-SIT, SSIT, and SSDT) were analyzed with Student's *t*-tests and Cohen's d and handedness with the chi-square test. The Pearson correlation-test was used to assess correlations between SSIT and SSDT and age and smoking in each group. Results are presented as a percentage or mean ± SD. Statistical significance threshold was set at *p* < 0.05.

### Analysis of fMRI Data

#### Olfactory Network Region of Interest Analysis

An ON region of interest mask was used in the fMRI analyses. The ON mask consisted of the piriform cortex, entorhinal cortex, anterior parahippocampal gyrus, hippocampus, amygdala, orbitofrontal cortex, insula, and thalamus and was created by combining the probabilistic maps of the Harvard–Oxford Structural Atlases and the Juelich Histological Atlas (part of FSL; http://www.fmrib.ox.ac.uk/fsl/fslview/atlas-descriptions.html#ho) as well as anatomical landmarks for the piriform cortex (34).

#### fMRI Analysis

Imaging data were analyzed using FSL 6.0.3 (Analysis Group, FMRIB, Oxford, UK). First, non-brain tissue was removed from the T1-weighted 3D images using BET 2 with robust center estimation (Brain Extraction Tool, FMRIB, Oxford, UK). The resulting images were transformed to the Montreal Neurological Institute (MNI) 1 × 1 × 1 mm^3^ template (Montreal Neurological Institute, Montreal, QC, Canada) non-linearly with FNIRT (FMRIB, Oxford, UK). The fMRI data were motion corrected using MCFLIRT with the median volume of each run as reference. Importantly, none of the subjects showed a mean relative root mean square displacement above 0.5 mm, the threshold in FSL for movement considered too severe to be corrected by MCFLIRT. Subsequently, each functional run was co-registered to the anatomical T1-weighted image before it was transformed into MNI space by using the transformation matrix obtained with the T1-weighted image. The functional data were smoothed with a 9-mm full-width at half-maximum Gaussian filter and temporally high-pass filtered with a cutoff time of 130 s.

Within the ON mask, voxelwise statistical analysis was performed using FEAT (FMRIB, Oxford, UK). For each subject, the two runs were analyzed separately (first level) and then combined within individuals using a fixed-effects GLM analysis (second level). Finally, effects across individuals were estimated by using separate GLM models and FLAME 1 (FMRIB's Local Analysis of Mixed Effects) (third level). At the first level, the explanatory variables were odor presentation (OI state blocks), scent removal (a 7.8 s period following odor presentation to ensure removal of scented air), and water baseline. At the third level, five GLM models investigated differences in activation within the ON using the contrast OI state > water baseline between the patients with aMCI/mAD and the control groups. The first model included one categorical variable for the aMCI/mAD and one categorical variable for the control group to evaluate group differences during the OI state. In the second model, age, sex, education, and brain parenchymal fraction (35, 36) were added as separate regressors. The parenchyma brain fraction was obtained from the T1weighted MPRAGE volume and estimated using FreeSurfer 6.0.0 (http://freesurfer.net/fswiki). One patient and one control were excluded from this analysis because their education level was missing, and two more patients were excluded because they did not pass the FreeSurfer quality assessment (https://surfer.nmr.mgh.harvard.edu/fswiki/QATools). In the third model, the average SSIT score was added as a separate regressor to the first model to investigate the effect of odor identification ability on activity within the ON. In the fourth model, the average SSDT score was added as a separate regressor to the first model to assess the impact of SSDT ability on activational differences between the aMCI/mAD and control group. The fifth model included the average MMSE score as a separate regressor to the first model to evaluate any association between MMSE score and brain activity during the OI state. Presence of an interaction between group and SSDT, SSIT, or MMSE performance on brain activity was investigated by splitting the score regressor into one regressor for aMCI/mAD and one for the control group. If no significant interaction was observed, the model without the interaction term was used. We choose to use the SSIT scores as covariate because of the larger score span for that test (16 possible correct points) compared to B-SIT and because it is the counterpart to the odor discrimination test (SSDT). An earlier study has shown a positive correlation between SSIT and B-SIT scores among aMCI/mAD patients (37). Unexpectedly, the planned event analysis (self-reported successful odor identification during fMRI) could not be performed due to too few events in both the patient and control groups. For model one, contrast OI state > water baseline was also investigated for each group separately. An independent two-sample *t*-test was used to investigate differences between the aMCI/mAD and control group, while one-sample *t*-tests were used to investigate the average effect for the patient or control group separately. Each voxel was thresholded using *Z* = 3.5 (*p* = 0.0005) to define contiguous clusters. The significance level of each cluster was then estimated from GRF theory using a corrected cluster threshold of *p* = 0.05.

## Results

### Demographics and Clinical Variables

All included participants had normal anterior rhinoscopy and normal sinuses and posterior nasal structures on MRI. The aMCI/mAD group was slightly older than the control group, but no significant differences were found in education level or smoking habits between the groups (Table 1). Sniffin' Sticks Test scores were significantly correlated with age (*r* = 0.436, *p* = 0.003) and smoking (*r* = 0.510, *p* < 0.0001) in both the aMCI/mAD group and control group.

**Table 1:** Characteristics of the control group and the aMCI/mAD group.

**Characteristics**	**Controls (*n* = 28)**	**aMCI/mAD (*n* = 17)**
Gender (female/male %)	47/53	53/47
Age (years), mean (SD)	67.4 (7.6)^*^	74.4 (6.5)
Education (years), mean (SD)	17.1 (3.5)	15.3 (2.6)
Daily smokers (%)	3.4%	6.3%
APOE4 genotype (% carriers 1–2 alleles) (*n* = 15/24)	20.0%	73.3%
CSF total amyloid beta, mean (SD) (*n* = 0/11)	-	575.8 (227.6)

*Significant differences using Student's t-test for control group compared to aMCI/mAD group*,

* *p < 0.005*.

*aMCI, amnestic mild cognitive impairment; APOE4, apolipoprotein E4; CSF, cerebrospinal fluid; mAD, Alzheimer's dementia of mild degree*.

Significantly more patients than controls were APOE4 carriers (Table 1). The CSF total amyloid beta from the 11 patients (seven aMCI and four mAD) undergoing lumbar puncture were in the range considered as indicating Alzheimer's disease (38) (Table 1).

Mini Mental Status Examination scores and performance of the cognitive tests were significantly lower in the aMCI/mAD group compared to those in the control group (Table 2). The aMCI group performed significantly slower on both TMT-A and TMT-B tests than the mAD group (Table 1).

**Table 2:** Performance of cognitive tests (mean ± SD) for the control group, combined aMCI/mAD group, and the aMCI and mAD groups separately.

	**Controls (*n* = 28)**	**aMCI/mAD group (*n* = 17)**	**aMCI group at MRI (*n* = 11)**	**mAD group at MRI (*n* = 6)**
MMSE (max 30)	28.7 ± 1.2	25.5 ± 2.5^**^	26.0 ± 1.6	24.7 ± 3.7
Ten-Word Test, total recall (max 30)	22.7 ± 3.6	12.5 ± 3.8^**^	12.6 ± 4.2	12.4 ± 2.9
Ten-Word Test, delayed recall (max 10)	8.1 ± 1.9	2.2 ± 1.7^**^	2.27 ± 1.8	2.0 ±1.6
Trail Making Test-A (s)	52.6 ± 21.1	64.7 ± 21.8^**^	72.5 ± 17.9	50.5 ± 24.5^*^
Trail Making Test-B (s)	104.1 ± 37.5	140.3 ± 51.8^**^	150.3 ± 58.3	122.8 ± 26.0^*^

*Significant differences using Student's t-test*,

* *p < 0.05*,

** *p < 0.0005*.

*aMCI, amnestic mild cognitive impairment; mAD, Alzheimer's dementia of mild degree; MMSE, Mini Mental Status Examination; s, seconds*.

There was no significant difference in handedness between the patient and control groups (Pearson chi square *p* = 0.55, df 13) (39).

### Post-scan Assessment of Olfaction Abilities

Compared to the control group, the aMCI/mAD group had significantly lower OI ability as determined with post-scan uncued OI and multiple-choice OI tests using the odors presented during scanning, B-SIT, and SSIT, as well as lower odor discrimination ability, as determined with SSDT (Table 3).

**Table 3:** Olfactory tests scores (mean ± SD) for the control group compared to aMCI/mAD group.

	**Controls**	**aMCI/mAD**	***p*-value**	**Cohen's *d***
1. **Clinical psychophysical tests**				
B-SIT (max 12) (*n* = 17/28)	9.5 ± 2.0	6.7 ± 2.6	**<0.0005**	1.25
SSIT (max 16) (*n* = 16/28)	12.8 ± 2.4	9.4 ± 3.0	**0.001**	1.25
SSDT (max 16) (*n* = 12/27)	9.7 ± 2.8	7.5 ± 3.0	0.099	0.76
2. **Post-scan OI-tests**				
Post-scan test free recall (max 10) (*n* = 16/27)	3.9 ± 2.1	2.0 ± 1.4	**0.004**	1.12
Post-scan test multiple choice (max 10) (*n* = 16/27)	8.0 ± 2.0	6.1 ± 2.0	**0.003**	0.97

*Significant differences using Student's t-test and Cohen's d for control group compared to aMCI/mild AD group. The number of patients and controls for each test is presented as n = patients/controls in the table*.

*aMCI, amnestic mild cognitive impairment; B-SIT, Brief Smell Identification Test; mAD, Alzheimer's dementia of mild degree; OI, odor identification; SSIT, Sniffin' Sticks Identification Test; SSDT, Sniffin' Sticks Discrimination Test. Bold font indicates statistically significant group differences*.

### Olfactory fMRI

**Figure 2:**
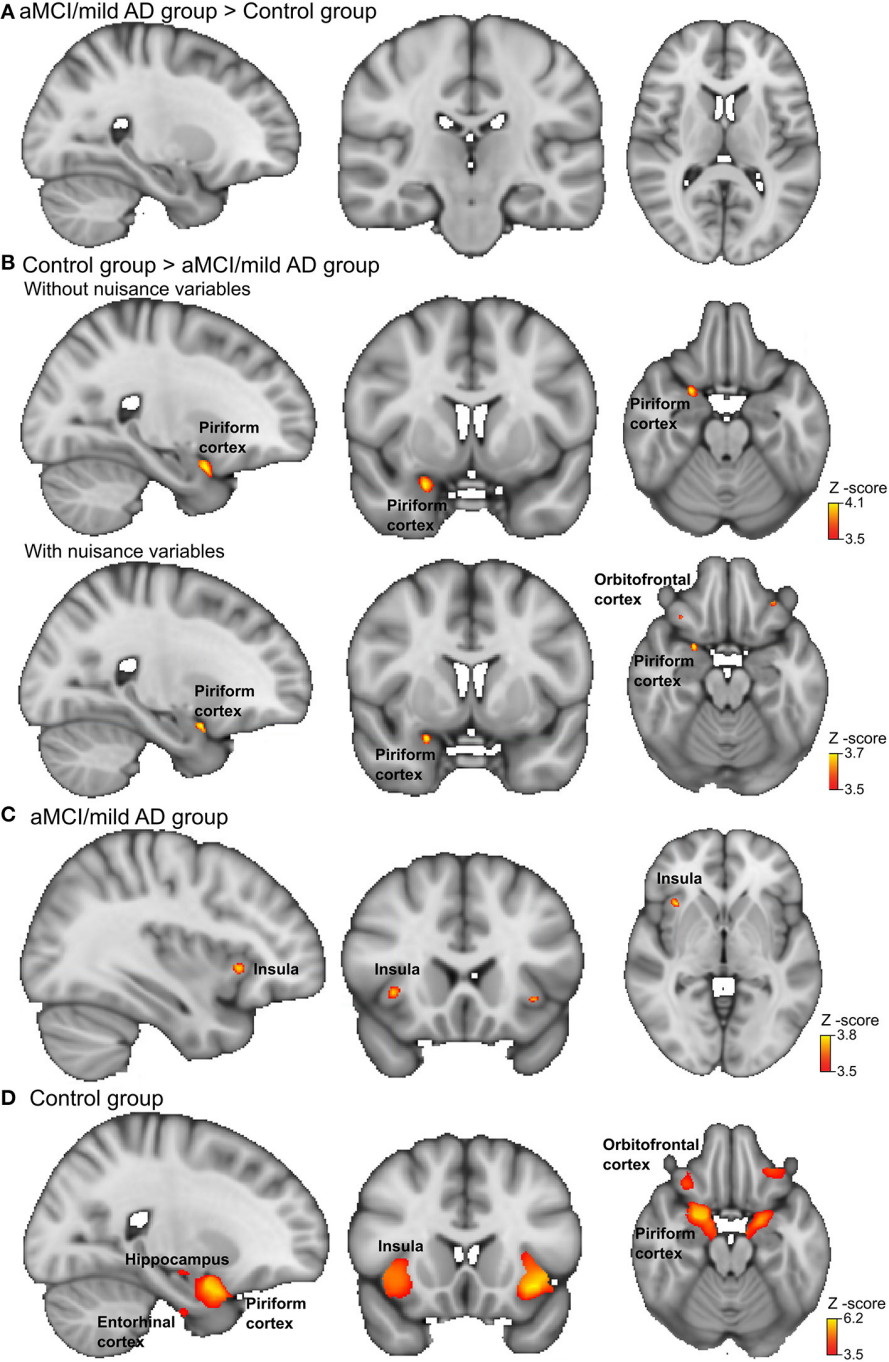
Brain activation during odor identification for amnestic mild cognitive impairment (aMCI)/Alzheimer's dementia of mild degree (mAD) patients and controls. Voxels in the olfactory cortices that showed increased activation during passive smelling for **(A)** aMCI/mAD patients > Controls, **(B)** Controls > aMCI/mAD patients, aMCI/mAD patients in upper row and with the nuisance variables age, sex, education, and brain parenchymal fraction in the lower row. **(C)** aMCI/mAD group and **(D)** Control group. The analysis was carried out using an olfactory network region of interest mask and a corrected cluster threshold of p = 0.05 (see section Materials and Methods). The “x=” in the lower left corner of each brain image indicates the position in the Montreal Neurological Institute (MNI) space. For more details on activation locations, see Table 4; for associations with olfactory test scores and cognition, see Table 5.

During OI fMRI, the aMCI/mAD group had reduced activation of the right piriform cortex with the peak located to the anterior subdivision compared to the control group (Figure 2 and Table 4) (Z-max = 4.1, cluster size = 297). Importantly, the reduced activation in the right piriform cortex for the aMCI/mAD group persisted after controlling for age, sex, education, and brain parenchymal fraction. Two smaller clusters of increased activation in orbitofrontal cortex also appeared after correction. The difference in activation in the piriform cortex between the aMCI/mAD and the control group was reduced when controlling for SSIT performance (Piriform: Z-max = 3.8, cluster size = 57) and disappeared when controlling for SSDT score. Controlling for MMSE score affected the group difference in piriform cortex activity only to a minor extent (Piriform: Z-max = 3.9, cluster size = 169). There was no interaction effect between group and SSIT, SSDT, or MMSE score on brain activity. In the combined group, only SSDT score was associated with fMRI activity located in the right piriform cortex, while no associations were present between SSIT or MMSE scores and fMRI activity (Table 5).

**Table 4:** Location of peak brain activations in MNI space during odor identification in the control group and aMCI/mAD groups.

**Brain region**	**Hemisphere**	**Cluster nr**.	**Cluster size**	***Z*-value (max)**	**X**	**Y**	**Z**
***aMCI/mAD*** **>** **Controls**							
*na*							
***aMCI/mAD*** **>** **Controls, with nuisance variables**							
*na*							
**Controls** **>** ***aMCI/mAD***							
Piriform cortex	R	1	297	4.13	24	6	−22
**Controls** **>** ***aMCI/mAD*****, with nuisance variables**							
Piriform cortex	R	1	86	3.73	24	5	−20
Orbitofrontal cortex	L	2	37	3.69	−31	36	−18
Orbitofrontal cortex	R	3	23	3.61	34	25	−18
***aMCI/mAD group***							
Insula, anterior	R	1	126	3.79	35	19	−1
Insula, anterior	L	2	42	3.71	−35	19	−5
**Control group**							
Piriform cortex	R	1	11,209	5.86	23	3	−22
Insula, anterior	R	1	11,209	4.91	38	19	−7
Entorhinal cortex	R	1	11,209	4.72	16	−7	−17
Orbitofrontal cortex	R	1	11,209	4.69	48	20	−9
Insula, anterior	L	2	5,926	6.24	−34	18	−9
Orbitofrontal cortex	L	2	5,926	4.91	−39	26	−1
Orbitofrontal cortex	L	2	5,926	4.44	−32	33	−22
Piriform cortex	L	3	2,403	5.67	−19	0	−22
Hippocampus, anterior	L	3	2,403	4.4	−10	−9	−20
Thalamus, prefrontal	L	4	1,756	4.99	−9	−3	1
Thalamus, temporal	L	4	1,756	4.94	−7	−3	−3
Thalamus, temporal	R	4	1,756	4.46	0	−17	3
Thalamus, prefrontal	R	4	1,756	4.19	4	−18	9
Insula, posterior	L	5	210	3.81	−39	−5	8
Thalamus, parietal	L	6	192	3.9	−12	−30	−4
Thalamus, sensory	L	6	192	3.83	−13	−23	−5
Thalamus, motor	L	6	192	3.8	−11	−24	−4
Perirhinal cortex	L	8	27	3.6	−27	−12	−42
Subcallosal cortex	L	9	17	3.77	−7	12	−22

*The analysis was carried out using an olfactory network mask (see section Materials and Methods for structures included) and a corrected cluster threshold of p = 0.05. X, Y, and Z are coordinates of peak activation in MNI coordinates using the 1-mm template. Only clusters that were larger than or equal to one functional voxel were reported, and up to five local maxima were reported for each cluster. The nuisance variables included age, sex, education, and brain parenchymal fraction. R, right; L, left. See Figure 2 for brain maps*.

*aMCI, amnestic mild cognitive impairment; mAD, Alzheimer's dementia of mild degree; MNI, Montreal Neurological Institute*.

**Table 5:** Location of brain activations during OI fMRI associated with olfactory test scores and Mini Mental Status Examination score.

**Brain region**	**Hemisphere**	**Cluster nr**.	**Cluster size**	***Z*-value (max)**	**X**	**Y**	**Z**
**SSDT**							
Piriform cortex	R	1	10	3.66	12	−7	−21
**SSIT**							
na							
**MMSE**							
na							

The GLM analyses were carried out within the olfactory network mask (see section Materials and Methods for structures included) and a corrected cluster threshold of p = 0.05. X, Y, and Z are coordinates of peak activation in MNI coordinates using the 1-mm template. Since there were no group *test performance interactions, the associations are assessed across both groups. Only clusters that were larger than or equal to one functional voxel were reported, and up to five local maxima were reported for each cluster. R, right; L, left; MMSE, Mini Mental Status Examination; MNI, Montreal Neurological Institute; OI, odor identification; SSDT, Sniffin' Sticks Discrimination Test; SSIT, Sniffin' Sticks identification test.

In the separate group analysis, the aMCI/mAD group displayed activation above the statistical threshold only in the bilateral anterior insula (Figure 2). No activity within the ON correlated with CSF total-amyloid beta in the aMCI/mAD group. In the control group, increased activation was present within all ON regions during OI fMRI (Table 4 and Figure 2).

## Discussion

In this OI fMRI study combined with psychophysical tests of olfactory functions, the aMCI/mAD group recruited neuronal resources in the piriform cortex significantly less and performed markedly poorer on all OI tests and the odor discrimination test than the control group. However, we did not find the expected lower brain activity in the entorhinal cortex or amygdala in the aMCI/mAD compared to the control group. Within the aMCI/mAD group, only insula activity was detected within the ON, in line with this region being affected later in the course of AD than the temporal and frontal brain regions. In the control group, on the other hand, all regions of the ON were strongly activated by the OI fMRI task, verifying the validity of the paradigm in activating the ON.

The piriform cortex activity was associated with OI impairment, as the difference between the aMCI/mAD and control groups became more restricted when controlling for SSIT. Nevertheless, the piriform cortex activity was mainly driven by odor discrimination ability, since the group difference disappeared completely when controlling for SSDT. The peak of the activation difference was located in the anterior piriform cortex, which is the main recipient of afferents from the olfactory bulb. The anterior piriform cortex also has extensive internal as well as external (e.g., with the orbitofrontal cortex) reciprocal connections (40, 41). The primary role of the anterior piriform cortex is odor discrimination and identification (42–44) as clearly reflected by the current results. Reduced piriform cortex activity has been reported in previous neuroimaging studies in patients with MCI and AD using different olfactory perception tasks (19, 21, 22, 45). These studies focused on metrics extracted from regions of interest analyses in the piriform or primary olfactory cortex, making direct comparison with these studies difficult. The present study extends these findings by performing OI fMRI in carefully selected patients using a variety of odors, which were dispensed with an automated olfactometer, combined with preprocessing and statistical analysis of the fMRI activity using a strict cluster-defining threshold as recommended. Taken together, the previous studies together with the current results demonstrate lower activity in the piriform cortex to olfactory stimuli from the first symptomatic stage of AD to late AD dementia. This is consistent with tau pathology being present in the piriform cortex from the very early symptomatic stage of AD (13, 46).

The reduced piriform activity in the aMCI/mAD group compared to the control group could originate from altered input to the piriform cortex as well as from local pathology. AD-related tau pathology is present in the olfactory bulb before it is found in the piriform cortex and could impair upstream activation of the piriform cortex (4, 13). Still, it appears unlikely that the lower activity in the piriform cortex in the aMCI/mAD group was caused mainly by reduced input from the olfactory bulb, as the activity difference was associated with odor identification (SSIT) and odor discrimination (SSDT) abilities, functions localized to the piriform cortex and not the olfactory bulb. Furthermore, β-amyloid plaques in the orbitofrontal cortex at the time of the first AD-related symptoms/MCI (4, 13) could also influence activity in the piriform cortex. The reciprocal piriform–orbitofrontal network is involved in both odor discrimination and identification (47) and might be disrupted both by local AD pathology in piriform and orbitofrontal cortices and/or through impaired downstream activation of the orbitofrontal cortex due to, for instance, a primary deficit in processing of olfactory stimuli in the piriform cortex in the aMCI/mAD group. The presence of significantly higher activity in the control group in two small clusters in the orbitofrontal cortex after correction for age, sex, education, and brain parenchymal fraction provides additional evidence for aberrant reciprocal piriform–orbitofrontal activity in the aMCI/mAD group. Taken together, the OI impairment in patients with MCI and AD likely arises from local pathology that affects stimulus processing, amplified by the disruption of network activity. Both aberrant downstream signaling from the piriform cortex and altered downstream processing of olfactory information could explain the lack of the expected differences in fMRI activity in the entorhinal cortex and amygdala. These regions are affected by tau pathology even earlier than the piriform cortex and before onset of aMCI/mAD symptoms.

Indeed, the lack of other group differences in activity during OI fMRI suggested highly variable brain activity in these regions in the aMCI/mAD group. A network affected by regional pathology within a network of regions would give rise to such variability. The presence of such variability could explain the need for uncorrected statistical thresholding in previous fMRI studies on olfaction in MCI/AD. The two previous olfactory fMRI studies in participants with MCI/AD reporting uncorrected voxel-based whole-brain activation show low overall brain activity and fewer and varying brain regions activated in MCI/AD (22).

The lack of an interaction between group and SSIT, SSDT, and MMSE scores on piriform activity demonstrated similar functional roles of the piriform cortex in the aMCI/mAD and control groups. Within the aMCI/mAD group, the only consistent activity during OI fMRI was located in the anterior insula, which is known to be affected later by AD-related pathology, at a time when dementia is well-established, compared to the other areas within the ON (4, 13, 17). CSF total β-amyloid levels were not associated with the anterior insula activity in the aMC/mAD group, neither were SSIT, SSDT, or MMSE scores. The lack of a group difference in insula activity and the similar location of the peak activation suggest that the anterior insula is involved in a similar manner during OI fMRI in the aMCI/AD and control groups. The insula activity in the aMCI/mAD and control groups was located in the same coordinates as in healthy young adults during OI fMRI (23). Extensive functional connections have been reported between piriform cortex and anterior insula in healthy adults, supporting the importance of insula in olfaction (48). Given the aberrant activity in the piriform cortex, it is possible that the activity observed in anterior insula is not related to olfaction *per se*. The lack of associations between insula activity and SSIT and SSDT scores supports this interpretation. Insula has important functions in cognitive effort, and the same insula region activated in the MCI/mAD and control groups during OI fMRI has previously been shown to have a higher fMRI signal during high compared to low cognitive effort conditions (49–51). Spontaneous OI is cognitively challenging, and the anterior insula activity may represent cognitive control efforts. Mini Mental Status Examination scores were not associated with the insula activity, but since these scores do not reflect effort, this observation does not rule out the possibility of insula activity representing cognitive effort during OI fMRI. The anterior insula is highly connected to several brain regions including all other ON regions (49), has no or limited AD pathology in the MCI and early AD phases (46, 52), and as such may receive sufficient input from various sources within the ON and other brain regions connected to, for instance, cognitive control to generate a consistent fMRI signal similar to that in the control group.

The aMCI/mAD group in this study had a higher mean MMSE score than in a previous fMRI study on olfaction in MC/AD (21). They still scored significantly lower than the control group on all psychophysical tests, with the largest effect sizes found for the OI tests, in agreement with the literature (5, 7). On average, the aMCI/mAD group scored about 30% lower on the OI tests while about 20% lower on the odor discrimination test, reflecting OI tests' superior ability to differentiate between aMCI/mAD and healthy elderly (53). The performance of the aMC/mAD group on SSIT was similar to that reported in Swedish and German patients with aMCI/mAD (54), lending credence to the generalizability of the current results across aMCI/mAD populations. As expected, the percentage of carriers of one or two APOE4 alleles was significantly higher in the aMCI/mAD group than among controls. Harboring APOE4 alleles affects olfaction even in healthy older adults (9), and the group difference in brain activity could be enhanced by this imbalance. Nevertheless, an APOE4 imbalance will be present in studies of aMCI/AD compared to health elderly due to the importance of APOE4 for AD risk (55).

There are several limitations of this study. Firstly, we designed a very simple OI fMRI paradigm based on experience with difficulties getting older adults to perform more advanced olfactory fMRI paradigms. Despite the simple task design and pre-scan training, the participants in both the aMCI/mAD and control groups did not press the button to signal successful odor identification during scanning as younger adults do (23) and the older adult pilot data indicated. The number of successful spontaneous OI during fMRI was so low that it was impossible to perform the planned event analysis. Since participants in both groups were able to spontaneously identify odors presented outside the scanner, it could be that the fMRI setting made them more cautious and/or insecure with regard to their own OI success or that they forgot to press the response buttons when they were not prompted by an examiner. We therefore analyzed activation associated with the OI state, i.e., the bloc where participants attempted to identify odors. Secondly, the control group was slightly younger than the aMCI/AD group. Given that the fMRI analysis correcting for age, sex, education, and brain parenchymal fraction was quite similar to the analysis without these covariates, the age difference between the two groups should not have affected the fMRI results (56). There was no significant difference in the frequency of smokers between the two groups, and as such, the effect of being a smoker on OI performance should be limited. Thirdly, the sample size was modest, and we were not able to include enough patients to reach the estimated group size due to MRI compatibility issues and clinical characteristics uncovered during scanning or in the follow-up period. We were still able to uncover significant group differences even with fewer participants in the patient group though. Nevertheless, by including a larger group of patients, we might have uncovered more details especially with regard to differences in brain activity in the regions with high fMRI signal variability in the patient group, such as the medial temporal lobe. Calculating sample size is difficult in fMRI studies, but based on previous fMRI studies in MCI/dementia and other clinical populations, between 12 and 23 participants is most common in clinical fMRI (57). With small samples, both type I and type II errors can be present, but the statistical approach implemented here has been shown to limit the inflated error rate for a two-sample *t*-test, even with only 10 subjects in each group (57). Importantly, after correcting for age, sex, education, and brain parenchymal fraction, the main group difference in activity in the piriform cortex remained significant (even though the patient group size was reduced with three cases due to missing data), demonstrating a robust group difference. A fourth limitation of this study was the use of the ON region of interest mask, which does not allow for uncovering group differences in fMRI activity in brain regions outside the ON.

A strength of the study is that all aMCI patients included were followed prospectively and clinically diagnosed as converted to AD or not. Moreover, the fMRI image analysis approaches and statistical thresholding were rigorously adhering to best practices, including a strict corrected threshold that correctly controls the family-wise error rate (57).

## Conclusion

Our findings show that during OI fMRI, patients with aMCI/mAD recruited the piriform cortex significantly less than the controls, and this activity was strongly associated with odor discrimination ability and to a lesser extent OI. The lack of consistent activity in the other ON structures in the aMCI/mAD group suggested large variability in activity due to differences in local AD-related pathology accompanied by aberrant downstream and reciprocal signaling within the regions of the ON. The reduced activity in the piriform cortex and normal activity in the anterior insula in the aMCI/mAD group likely reflect the presence of AD-related pathology in the piriform cortex but not in the insula, in accordance with the AD stages of the included patients. OI tests thus reflect AD pathology in olfactory structures.

## Data Availability Statement

The raw data supporting the conclusions of this article will be made available by the authors, without undue reservation.

## Ethics Statement

The studies involving human participants were reviewed and approved by REC Central—Secretariat. The patients/participants provided their written informed consent to participate in this study.

## Author Contributions

All authors listed have made a substantial, direct and intellectual contribution to the work, and approved it for publication.

## Conflict of Interest

The authors declare that the research was conducted in the absence of any commercial or financial relationships that could be construed as a potential conflict of interest.

## Abbreviations

OI: odor identification
ON: olfactory network
aMCI: amnestic mild cognitive impairment
mAD: Alzheimer's dementia of mild degree
fMRI: functional magnetic resonance imaging
BOLD: blood oxygen level-dependent
B-SIT: Brief Smell Identification Test
SSIT: Sniffin' Sticks Test
SSDT: Sniffin' Sticks Discrimination Test.

## References

[1] Rahayel S Frasnelli J Joubert S. The effect of Alzheimer's disease and Parkinson's disease on olfaction: a meta-analysis. Behav Brain Res. (2012) 231:60–74. 10.1016/j.bbr.2012.02.047 22414849

[2] Ruan Y Zheng XY Zhang HL Zhu W Zhu J. Olfactory dysfunctions in neurodegenerative disorders. J Neurosci Res. (2012) 90:1693–700. 10.1002/jnr.23054 22674288

[3] Mesholam RI Moberg PJ Mahr RN Doty RL. Olfaction in neurodegenerative disease: a meta-analysis of olfactory functioning in Alzheimer's and Parkinson's diseases. Arch Neurol. (1998) 55:84–90. 10.1001/archneur.55.1.84 9443714

[4] Silva MME Mercer PBS Witt MCZ Pessoa RR. Olfactory dysfunction in Alzheimer's disease systematic review and meta-analysis. Dement Neuropsychol. (2018) 12:123–32. 10.1590/1980-57642018dn12-020004 29988355 PMC6022986

[5] Djordjevic J Jones-Gotman M De Sousa K Chertkow H. Olfaction in patients with mild cognitive impairment and Alzheimer's disease. Neurobiol Aging. (2008) 29:693–706. 10.1016/j.neurobiolaging.2006.11.014 17207898

[6] Westervelt HJ Bruce JM Coon WG Tremont G. Odor identification in mild cognitive impairment subtypes. J Clin Exp Neuropsychol. (2008) 30:151–6. 10.1080/13803390701287408 18938667

[7] Suzuki Y Yamamoto S Umegaki H Onishi J Mogi N Fujishiro H. Smell identification test as an indicator for cognitive impairment in Alzheimer's disease. Int J Geriatr Psychiatry. (2004) 19:727–33. 10.1002/gps.1161 15290695

[8] Schubert CR Carmichael LL Murphy C Klein BE Klein R Cruickshanks KJ. Olfaction and the 5-year incidence of cognitive impairment in an epidemiological study of older adults. J Am Geriatr Soc. (2008) 56:1517–21. 10.1111/j.1532-5415.2008.01826.x 18662205 PMC2587240

[9] Olofsson JK Larsson M Roa C Wilson DA Jonsson Laukka E. Interaction between odor identification deficit and APOE4 predicts 6-year cognitive decline in elderly individuals. Behav Genet. (2020) 50:3–13. 10.1007/s10519-019-09980-9 31760549 PMC6941999

[10] Devanand DP Liu X Cohen H Budrow J Schupf N Manly J. Long-term test-retest reliability of the UPSIT in cognitively intact older adults. Chem Senses. (2019) 44:365–9. 10.1093/chemse/bjz025 31111142 PMC7357246

[11] Saive AL Royet JP Plailly J. A review on the neural bases of episodic odor memory: from laboratory-based to autobiographical approaches. Front Behav Neurosci. (2014) 8:240. 10.3389/fnbeh.2014.00240 25071494 PMC4083449

[12] Doty RL editor. Handbook of Olfaction and Gustation. 2nd ed New York, NY: Marcel Dekker (2003).

[13] Braak H Del Trecidi K. Neuroanatomy and pathology of sporadic Alzheimer's disease. Adv Anat Embryol Cell Biol. (2015) 215:1–162. 10.1007/978-3-319-12679-1 25920101

[14] Chu CC Tranel D Damasio AR Van Hoesen GW. The autonomic-related cortex: pathology in Alzheimer's disease. Cereb Cortex. (1997) 7:86–95. 10.1093/cercor/7.1.86 9023436

[15] Arnold SE Hyman BT Van Hoesen GW. Neuropathologic changes of the temporal pole in Alzheimer's disease and Pick's disease. Arch Neurol. (1994) 51:145–50. 10.1001/archneur.1994.00540140051014 8304839

[16] Kromer Vogt LJ Hyman BT Van Hoesen GW Damasio AR. Pathological alterations in the amygdala in Alzheimer's disease. Neuroscience. (1990) 37:377–85. 10.1016/0306-4522(90)90408-V 2133349

[17] Oliveira FA Cellular Mechanisms in Alzheimer's Disease. Sharjah: Bentham Science Publishers (2018). p. 237.

[18] Waldemar G Dubois B Emre M Georges J McKeith IG Rossor M. Recommendations for the diagnosis and management of Alzheimer's disease and other disorders associated with dementia: EFNS guideline. Eur J Neurol. (2007) 14:e1–26. 10.1111/j.1468-1331.2006.01605.x 17222085

[19] Vasavada MM Martinez B Wang J Eslinger PJ Gill DJ Sun X. Central olfactory dysfunction in Alzheimer's disease and mild cognitive impairment: a functional MRI study. J Alzheimers Dis. (2017) 59:359–68. 10.3233/JAD-170310 28671131

[20] Vasavada MM Wang J Eslinger PJ Gill DJ Sun X Karunanayaka P. Olfactory cortex degeneration in Alzheimer's disease and mild cognitive impairment. J Alzheimers Dis. (2015) 45:947–58. 10.3233/JAD-141947 25633674

[21] Wang J Eslinger PJ Doty RL Zimmerman EK Grunfeld R Sun X. Olfactory deficit detected by fMRI in early Alzheimer's disease. Brain Res. (2010) 1357:184–94. 10.1016/j.brainres.2010.08.018 20709038 PMC3515873

[22] Zhang H Ji D Yin J Wang Z Zhou Y Ni H. Olfactory fMRI activation pattern across different concentrations changes in Alzheimer's disease. Front Neurosci. (2019) 13:786. 10.3389/fnins.2019.00786 31417348 PMC6682702

[23] Kjelvik G Evensmoen HR Brezova V Haberg AK. The human brain representation of odor identification. J Neurophysiol. (2012) 108:645–57. 10.1152/jn.01036.2010 22539820

[24] McKhann G Drachman D Folstein M Katzman R Price D Stadlan EM. Clinical diagnosis of Alzheimer's disease: report of the NINCDS-ADRDA Work Group under the auspices of Department of Health and Human Services Task Force on Alzheimer's Disease. Neurology. (1984) 34:939–44. 10.1212/WNL.34.7.939 6610841

[25] Petersen RC Smith GE Waring SC Ivnik RJ Tangalos EG Kokmen E. Mild cognitive impairment: clinical characterization and outcome. Arch Neurol. (1999) 56:303–8. 10.1001/archneur.56.3.303 10190820

[26] Winblad B Palmer K Kivipelto M Jelic V Fratiglioni L Wahlund LO. Mild cognitive impairment–beyond controversies, towards a consensus: report of the International Working Group on Mild Cognitive Impairment. J Intern Med. (2004) 256:240–6. 10.1111/j.1365-2796.2004.01380.x 15324367

[27] Fillenbaum GG van Belle G Morris JC Mohs RC Mirra SS Davis PC. Consortium to Establish a Registry for Alzheimer's Disease (CERAD): the first twenty years. Alzheimers Dement. (2008) 4:96–109. 10.1016/j.jalz.2007.08.005 18631955 PMC2808763

[28] Oldfield RC. The assessment and analysis of handedness: the Edinburgh inventory. Neuropsychologia. (1971) 9:97–113. 10.1016/0028-3932(71)90067-4 5146491

[29] Olman CA Davachi L Inati S. Distortion and signal loss in medial temporal lobe. PLoS ONE. (2009) 4:e8160. 10.1371/journal.pone.0008160 19997633 PMC2780716

[30] Popp R Sommer M Muller J Hajak G. Olfactometry in fMRI studies: odor presentation using nasal continuous positive airway pressure. Acta Neurobiol Exp (Wars). (2004) 64:171–6. 15366250 10.55782/ane-2004-1503

[31] Lorig TS Elmes DG Zald DH Pardo JV. A computer-controlled olfactometer for fMRI and electrophysiological studies of olfaction. Behav Res Methods Instrum Comput. (1999) 31:370–5. 10.3758/BF03207734 10495824

[32] Lowen SB Lukas SE. A low-cost, MR-compatible olfactometer. Behav Res Methods. (2006) 38:307–13. 10.3758/BF03192782 16956107 PMC1602106

[33] Vigouroux M Bertrand B Farget V Plailly J Royet JP. A stimulation method using odors suitable for PET and fMRI studies with recording of physiological and behavioral signals. J Neurosci Methods. (2005) 142:35–44. 10.1016/j.jneumeth.2004.07.010 15652615

[34] Pereira PMG Insausti R Artacho-Pérula E Salmenperä T Kälviäinen R Pitkänen A. MR volumetric analysis of the piriform cortex and cortical amygdala in drug-refractory temporal lobe epilepsy. Am J Neuroradiol. (2005) 26:319–32. 15709130 PMC7974111

[35] Rudick RA Fisher E Lee JC Simon J Jacobs L. Use of the brain parenchymal fraction to measure whole brain atrophy in relapsing-remitting MS. Multiple Sclerosis Collaborative Research Group. Neurology. (1999) 53:1698–704. 10.1212/WNL.53.8.1698 10563615

[36] Vagberg M Granasen G Svenningsson A. Brain parenchymal fraction in healthy adults-a systematic review of the literature. PLoS ONE. (2017) 12:e0170018. 10.1371/journal.pone.0170018 28095463 PMC5240949

[37] Kjelvik G Saltvedt I White LR Stenumgard P Sletvold O Engedal K. The brain structural and cognitive basis of odor identification deficits in mild cognitive impairment and Alzheimer's disease. BMC Neurol. (2014) 14:168. 10.1186/s12883-014-0168-1 25154749 PMC4236673

[38] Knapskog AB Eldholm RS Braekhus A Engedal K Saltvedt I. Factors that influence the levels of cerebrospinal fluid biomarkers in memory clinic patients. BMC Geriatr. (2017) 17:210. 10.1186/s12877-017-0611-4 28893185 PMC5594466

[39] Lübke K Gottschlich M Gerber J Pause BM Hummel T No effects of handedness on passive processing of olfactory stimuli: An fMRI study. Chemosens Percept. (2012) 5:22–6. 10.1007/s12078-011-9115-3

[40] Illig KR Projections from orbitofrontal cortex to anterior piriform cortex in the rat suggest a role in olfactory information processing. J Comp Neurol. (2005) 488:224–31. 10.1002/cne.20595 15924345 PMC1360190

[41] Roesch MR Stalnaker TA Schoenbaum G. Associative encoding in anterior piriform cortex versus orbitofrontal cortex during odor discrimination and reversal learning. Cereb Cortex. (2007) 17:643–52. 10.1093/cercor/bhk009 16699083 PMC2396586

[42] Cattarelli M Astic L Kauer JS. Metabolic mapping of 2-deoxyglucose uptake in the rat piriform cortex using computerized image processing. Brain Res. (1988) 442:180–4. 10.1016/0006-8993(88)91449-7 3359252

[43] Illig KR Haberly LB. Odor-evoked activity is spatially distributed in piriform cortex. J Comp Neurol. (2003) 457:361–73. 10.1002/cne.10557 12561076

[44] Ojima H Mori K Kishi K. The trajectory of mitral cell axons in the rabbit olfactory cortex revealed by intracellular HRP injection. J Comp Neurol. (1984) 230:77–87. 10.1002/cne.902300107 6096415

[45] Kareken DA Doty RL Moberg PJ Mosnik D Chen SH Farlow MR. Olfactory-evoked regional cerebral blood flow in Alzheimer's disease. Neuropsychology. (2001) 15:18–29. 10.1037/0894-4105.15.1.18 11216885

[46] Braak H Braak E. Neuropathological stageing of Alzheimer-related changes. Acta Neuropathol. (1991) 82:239–59. 10.1007/BF00308809 1759558

[47] Critchley HD Rolls ET. Olfactory neuronal responses in the primate orbitofrontal cortex: analysis in an olfactory discrimination task. J Neurophysiol. (1996) 75:1659–72. 10.1152/jn.1996.75.4.1659 8727404

[48] Zhou G Lane G Cooper SL Kahnt T Zelano C. Characterizing functional pathways of the human olfactory system. Elife. (2019) 8:e47177. 10.7554/eLife.47177 31339489 PMC6656430

[49] Uddin LQ Nomi JS Hebert-Seropian B Ghaziri J Boucher O. Structure and function of the human insula. J Clin Neurophysiol. (2017) 34:300–6. 10.1097/WNP.0000000000000377 28644199 PMC6032992

[50] Daly S Thai J Belkhiria C Langley C Blanche AL de Marco G. Temporal deployment of attention by mental training: an fMRI study. Cogn Affect Behav Neurosci. (2020) 20:669–83. 10.3758/s13415-020-00795-4 32458391

[51] Aben B Buc Calderon C Van den Bussche E Verguts T. Cognitive effort modulates connectivity between dorsal anterior cingulate cortex and task-relevant cortical areas. J Neurosci. (2020) 40:3838–48. 10.1523/JNEUROSCI.2948-19.2020 32273486 PMC7204076

[52] Fjell AM McEvoy L Holland D Dale AM Walhovd KB Alzheimer's Disease Neuroimaging Initiative. What is normal in normal aging? Effects of aging, amyloid and Alzheimer's disease on the cerebral cortex and the hippocampus. Prog Neurobiol. (2014) 117:20–40. 10.1016/j.pneurobio.2014.02.004 24548606 PMC4343307

[53] Peters JM Hummel T Kratzsch T Lotsch J Skarke C Frolich L. Olfactory function in mild cognitive impairment and Alzheimer's disease: an investigation using psychophysical and electrophysiological techniques. Am J Psychiatry. (2003) 160:1995–2002. 10.1176/appi.ajp.160.11.1995 14594747

[54] Tahmasebi R Zehetmayer S Pusswald G Kovacs G Stogmann E Lehrner J. Identification of odors, faces, cities and naming of objects in patients with subjective cognitive decline, mild cognitive impairment and Alzheimer s disease: a longitudinal study. Int Psychogeriatr. (2019) 31:537–49. 10.1017/S1041610218001114 30236169

[55] Liu CC Liu CC Kanekiyo T Xu H Bu G. Apolipoprotein E and Alzheimer disease: risk, mechanisms and therapy. Nat Rev Neurol. (2013) 9:106–18. 10.1038/nrneurol.2012.263 23296339 PMC3726719

[56] Doty RL Shaman P Applebaum SL Giberson R Siksorski L Rosenberg L. Smell identification ability: changes with age. Science. (1984) 226:1441–3. 10.1126/science.6505700 6505700

[57] Eklund A Nichols TE Knutsson H. Cluster failure: why fMRI inferences for spatial extent have inflated false-positive rates. Proc Natl Acad Sci. (2016) 113:7900–5. 10.1073/pnas.1602413113 27357684 PMC4948312

